# Vestibular paroxysmia: Long-term clinical outcome after treatment

**DOI:** 10.3389/fneur.2022.1036214

**Published:** 2022-10-14

**Authors:** Chih-Chung Chen, Ting-Yi Lee, Hsun-Hua Lee, Yu-Hung Kuo, Anand K. Bery, Tzu-Pu Chang

**Affiliations:** ^1^Dizziness and Balance Disorder Center, Taipei Medical University–Shuang Ho Hospital, New Taipei City, Taiwan; ^2^Taipei Neuroscience Institute, Taipei Medical University, New Taipei City, Taiwan; ^3^Department of Neurology, School of Medicine, College of Medicine, Taipei Medical University, Taipei, Taiwan; ^4^Department of Research, Taichung Tzu Chi Hospital, Buddhist Tzu Chi Medical Foundation, Taichung, Taiwan; ^5^Division of Neurology, Department of Medicine, University of Ottawa, Ottawa, ON, Canada; ^6^Department of Neurology, Neuro-Medical Scientific Center, Taichung Tzu Chi Hospital, Buddhist Tzu Chi Medical Foundation, Taichung, Taiwan; ^7^Department of Neurology, School of Medicine, Tzu Chi University, Hualien, Taiwan

**Keywords:** dizziness, neurovascular compression, trigeminal neuralgia, typewriter tinnitus, vertigo, vestibular paroxysmia

## Abstract

**Objective:**

To study the long-term treatment outcome of vestibular paroxysmia (VP).

**Study design:**

Retrospective study.

**Setting:**

Tertiary referral hospital.

**Methods:**

We analyzed records of 29 consecutive patients who were diagnosed with VP and who were treated with VP-specific anticonvulsants for at least 3 months. Patients were followed for a minimum of 6 months. We recorded and assessed starting and target dosage of medications, time to achieve adequate therapeutic response, adverse effects, and the rates of short-term and long-term remission without medication.

**Results:**

All 29 patients were started on oxcarbazepine as first-line treatment, and 93.1% and 100% of patients reported good-to-excellent therapeutic response within 2 and 4 weeks, respectively. Three patients switched to other anticonvulsants at 3 months. At long-term follow-up (8–56 months), most (84.6%) oxcarbazepine-treated patients maintained good therapeutic response at doses between 300 and 600 mg/day. Eleven (37.9%) patients experienced complete remission without medication for more than 1 month, of which six (20.7%) had long-term remission off medication for more than 12 months. Nineteen (65.5%) patients had neurovascular compression (NVC) of vestibulocochlear nerve on MRI, but its presence or absence did not predict treatment response or remission.

**Conclusion:**

Low-dose oxcarbazepine monotherapy for VP is effective over the long term and is generally well-tolerated. About 20% of patients with VP in our study had long-term remission off medication.

## Introduction

Vestibular paroxysmia (VP) is a debilitating clinical condition characterized by brief episodes of spontaneous or positional vertigo. Similar to trigeminal neuralgia (TN), VP is felt to be caused by neurovascular compression (NVC) of the vestibular nerve near the root entry zone (1). Vascular compression leads to focal demyelination and subsequent ectopic/ephaptic discharges of the affected cranial nerves (CN5 in TN; CN8 in VP). As they share the same presumed pathophysiology, TN and VP characteristically show significant responses to carbamazepine or oxcarbazepine.

The consensus paper on VP published by the Classification Committee for International Classification of Vestibular Disorders (ICVD) in 2016 has been widely adopted as a gold standard of diagnosis (Table 1) (2). The actual incidence of VP is unknown, but one study found it represented 3.9% of vestibular diagnoses at a tertiary outpatient clinic (3). Although it is a treatable disorder, it is easy to overlook VP because it can be difficult to distinguish from other causes of episodic dizziness on history and physical exam alone.

**Table 1:** ICVD diagnostic criteria for definite vestibular paroxysmia (2).

**(A)**	**At least 10 attacks of spontaneous spinning or non-spinning vertigo**
**(B)**	**Duration < 1 min**
**(C)**	**Stereotyped phenomenology in a particular patient**
**(D)**	**Response to treatment with carbamazepine/oxcarbazepine**
**(E)**	**Not better accounted for by another diagnosis**

ICVD, International Classification of Vestibular Disorders.

Despite significant therapeutic response to carbamazepine/oxcarbazepine, detailed outcome after treatment, especially long-term treatment, remains unknown. Clinicians intuitively suppose that most patients need life-long medical control unless undergoing microvascular decompression surgery, which is thought to be the only means of true “cure.” In our clinical experience, however, after a period of medical control we have seen some patients discontinue medication and remain symptom-free. If it holds true that some patients of VP have short-term or long-term remission, discontinuation of carbamazepine/oxcarbazepine may be a valuable option for patients who have been symptom-free for a period (e.g., 6 months). In addition, if medical management effectively controls symptoms over the long term, surgical intervention may not be the exclusive choice for cure. It has been demonstrated that various periods of complete remission is a characteristic of TN (4, 5). It is worth investigating whether VP has a similar natural history.

To answer these questions, we reviewed consecutive patients diagnosed with VP and followed them longitudinally at our center, with particular emphasis on their presenting clinical features, therapeutic response, and most importantly, long-term outcomes.

## Materials and methods

### Dizziness registry system and ethics

The data from this study come from a well-curated institutional registry on all patients with dizziness (The Dizziness Registry System) based out of the Dizziness and Balance Disorder Center at Taipei Medical University, Shuang Ho Hospital in Taipei, Taiwan. All patients who have received medical services at the center since its founding in 2017 were included and their diagnoses were recorded according to International Classification of Diseases, 9th Edition, Clinical Modification (ICD-9-CM) or International Classification of Diseases, 10th Edition (ICD-10). A well-trained case manager constantly updates data entries of registered patients by chart review and telephone interview. There are ongoing monthly team conferences to inspect and analyze the data. The study protocol was approved by our institution's Joint Institutional Review Board (N202108060). The board granted a waiver for written informed consent because of the retrospective nature of the study.

### Patients

**Figure 1:**
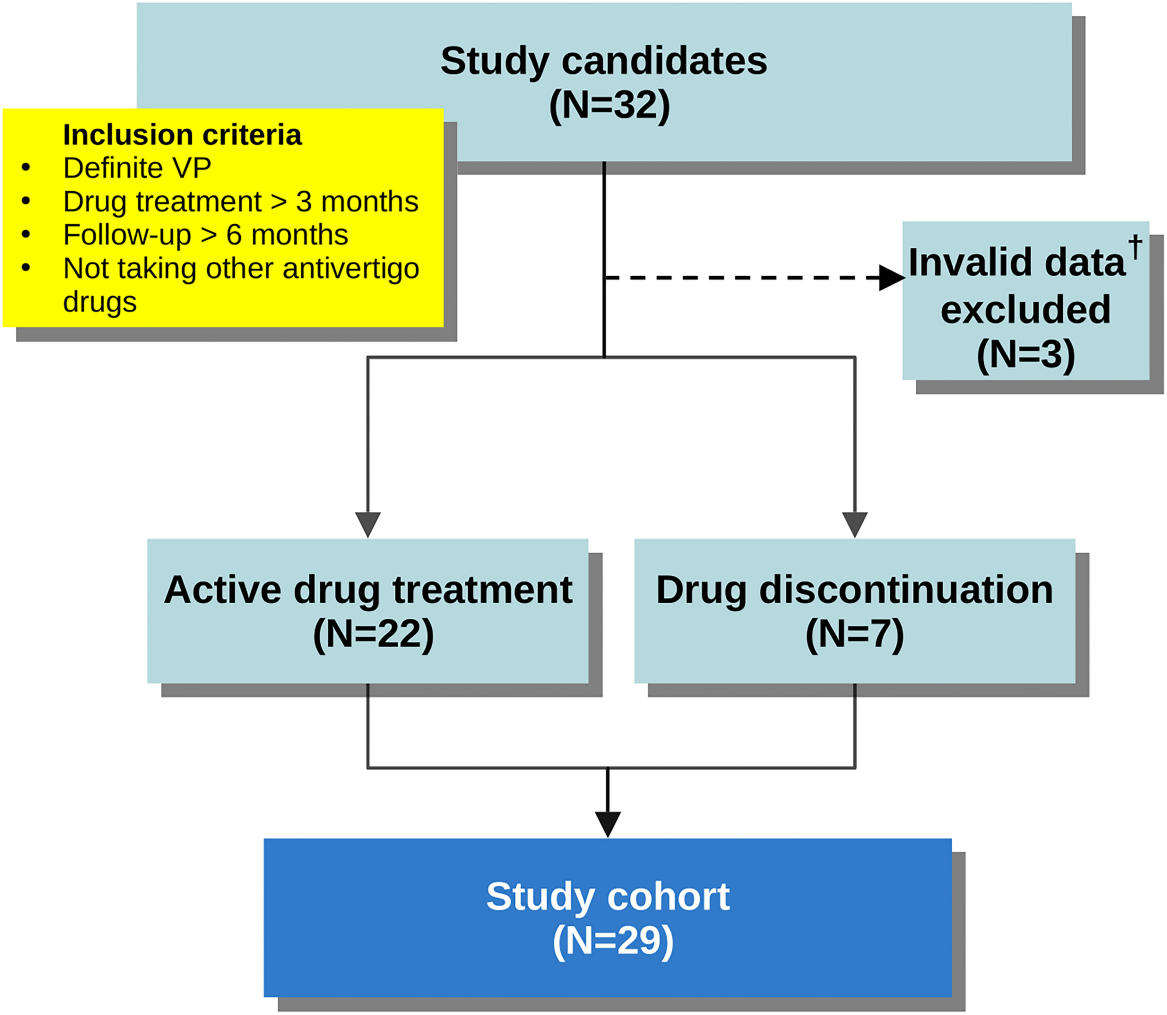
Flowchart demonstrating cohort derivation. ^†^Patient records that had not been updated in the last 3 months before study enrollment.

We retrospectively reviewed clinical information for all patients in our registry diagnosed with VP between 2017 and 2020. In order to investigate the long-term outcome and remission after medical treatment, we included the patients who fulfilled all of the following: (i) met ICVD criteria for “definite VP,” (ii) had received VP-specific anticonvulsants at least 3 months, (iii) had been continuously followed up for at least 6 months, and (iv) were not taking other anti-vertigo medications. In keeping with previous reports, carbamazepine, oxcarbazepine, gabapentin, pregabalin, lacosamide, and lamotrigine were all accepted as VP-specific anticonvulsants for the purposes of study enrollment (6–8). Figure 1 shows the flowchart for cohort derivation.

### Follow-up

All included patients were followed until July 31, 2021. At the conclusion of the study, records were reviewed and patients were classified into one of two groups: (i) those who remained on VP-specific anticonvulsants, or (ii) those who had discontinued said medications. Through chart review and telephone interview, we collected detailed information about initial symptoms, neurologic and neuro-otologic signs, medications, dosage, therapeutic response, and neuroimaging results. For the patients who had discontinued VP-specific medications, detailed information about the symptom-free duration, time of recurrence, and current use of non-specific vestibular suppressant medications were obtained. Patient records that had not been updated in the last 3 months were regarded as invalid and were excluded from this analysis.

### Treatment outcome

Since VP is a paroxysmal disorder, we defined therapeutic response as reduction in frequency of attacks. Change in attack frequency after treatment was dichotomized as follows: poor response (0–20% reduction), modest response (20–50% reduction), good response (50–80% reduction), and excellent response (80–100% reduction). In addition to vestibular attacks, episodes of concomitant paroxysmal cranial nerve disorders, including typewriter tinnitus, hemifacial spasm (HFS), TN, glossopharyngeal neuralgia, and inferior oblique myokymia, were also counted as attacks (9). “Stable dosage” was defined as the minimum dosage of the medication which could maintain “good” or “excellent” therapeutic response for at least 3 months. While there is no authoritative definition of remission for VP, we adopted the outcome measures used by a prospective study on TN (9). Short-term remission was defined as having continuous symptom-free periods, without use of anti-vertigo medications, for at least 1 month. Long-term remission was defined as continuous symptom-free periods of at least 12 months without medication.

### Statistical analysis

We recorded and analyzed the starting dosage of medications, therapeutic period of achieving good or excellent response for vestibular symptoms, stable dosage, durations of treatment, adverse effects, therapeutic responses for concomitant NVC disorders, and durations of remissions. The rates of short-term and long-term remissions without medication were calculated. We reported descriptive statistics and the cumulative remission rate after VP medical treatment. One-way ANOVA was used to compare the stable dosage of oxcarbazepine between the patients with and without NVC. Univariate logistic regression was used to determine if the duration of VP, age, the NVC in MRI, the concomitant paroxysmal cranial nerve disorders, the stable drug dosage or the duration of treatment can predict remission. Odds ratios and 95% confidence intervals (CI) were calculated. The statistical analysis was performed using SPSS v23 (Armonk, NY).

## Results

### Characteristics of patients

After excluding three invalid entries, 29 patients met study inclusion. Twenty-two (75.9%) patients were on active treatment at the end of follow-up. Seven (24.1%) patients were not on active treatment at the end of follow-up. The cohort comprised 19 males and 10 females, aged 44–79 and the follow-up periods ranged from 8 to 56 months (detailed information in Supplementary Table 1). The duration of symptoms before diagnosis of VP ranged from 1 month to 9 years. Fourteen patients had concomitant paroxysmal cranial nerve disorders—typewriter tinnitus in 13 (44.8%), HFS in 2 (6.9%), and TN in 2 (6.9%). Nystagmus was recorded in eight patients (27.6%), including ictal spontaneous nystagmus in 1 (3.4%), interictal spontaneous nystagmus in 1 (3.4%), head-shaking nystagmus in 3 (10.3%), hyperventilation-induced nystagmus in 3 (10.3%), and positional nystagmus in 3 (10.3%). MRI showed NVC in 19 (65.5%) patients, and 4 patients (13.8%) had prominent nerve distortion. Nine of the 14 patients with concomitant paroxysmal cranial nerve disorders had NVC ipsilateral to the lesion side (7/13 in typewriter tinnitus, 1/2 in HFS, and 1/2 in TN). The duration of treatment ranged from 4 to 56 months. Patient demographics, presenting symptoms, and treatment details of the study cohort are summarized in Table 2.

**Table 2:** Summary of the study cohort (*N* = 29).

**Age**		
	**Mean ± SD**	**64.7 ± 10.2**
	**Range**	**44–79**
**Sex (** ***n*** **)**		
	**Male**	**19**
	**Female**	**10**
**Duration of symptoms prior to index visit (months)**		
	**Mean ± SD**	**24 ± 26**
	**Range**	**1–108**
**Concomitantparoxysmal cranial nerve disorders (** ***n*** **)**		
	**Typewriter tinnitus**	**13**
	**Hemifacial spasm**	**2**
	**Trigeminal neuralgia**	**2**
**Medication (** ***n*** **)**		
	**Oxcarbazepine**	**26**
	**Pregabalin**	**2**
	**Gabapentin**	**1**
**Treatment duration (months)**		
	**Mean ± SD**	**23 ± 15**
	**Range**	**4–56**
**Follow-up duration (months)**		
	**Mean ± SD**	**30 ± 14**
	**Range**	**8–56**

### Therapeutic response for vestibular symptoms

All 29 patients were started on oxcarbazepine as first-line treatment, at a starting dosage of 300–600 mg/day. At 1-month follow-up, all patients achieved “good” or “excellent” therapeutic response (see definitions above). Specifically, 27 (93.1%) patients reported good-to-excellent therapeutic response within 2 weeks. At 3-month follow-up, 26 patients (89.7%) maintained good-to-excellent responses on oxcarbazepine while the other three patients (10.3%) switched to other anticonvulsants despite the initial good response of oxcarbazepine. Two of them switched to pregabalin due to intolerable side effects of oxcarbazepine (weakness and hyponatremia). One switched to gabapentin because the therapeutic effect of oxcarbazepine diminished.

At long-term follow-up, the same 26 patients continued on oxcarbazepine monotherapy and had satisfactory control. Among them, 15 patients remained at a stable dosage of 300 mg/day, one at 450 mg/day, six at 600 mg/day, two at 900 mg/day, and two at 1,200 mg/day. Most (22/26, 84.6%) of the patients had optimal response at doses between 300 and 600 mg/day. Two patients who had concomitant HFS required higher dosages (900–1,200 mg/day) to relieve their dizziness (see Table 3 for details on therapeutic response). The stable dosages were not significantly different between the patients with NVC and without NVC (mean dosage 485 vs. 500 mg, *p* = 0.90, One-way ANOVA).

**Table 3:** Oxcarbazepine regimen and efficacy in 26 VP patients.

**No**.	**Age**	**Sex**	**Concomitant cranial nerve disorders**	**Duration of follow-up (months)**	**Therapeutic response for dizziness**	**Weeks to optimal response**	**Stable dosage (mg/d)**	**Therapeutic response for concomitant cranial nerve disorders**
1	54	M	–	14	Excellent	1	300	N/A
2	65	M	Tinnitus	42	Excellent	1	300	Modest
3	71	F	Tinnitus	44	Excellent	1	600	Modest
4	76	M	–	50	Excellent	1	300	N/A
5	73	M	–	15	Excellent	4	300	N/A
6	57	M	Tinnitus	10	Excellent	2	900	Modest
7	69	M	Tinnitus	25	Excellent	2	300	Good
8	48	M	–	22	Excellent	2	300	N/A
9	57	F	Tinnitus	14	Excellent	2	450	Good
10	66	M	–	56	Excellent	1	600	N/A
12	51	M	HFS	38	Excellent	1	900	Poor
13	69	M	–	42	Excellent	2	300	N/A
14	74	M	–	33	Excellent	1	300	N/A
15	73	F	Tinnitus	14	Excellent	1	600	Excellent
16	75	M	–	46	Excellent	2	300	N/A
17	79	F	Tinnitus, HFS, TN	8	Good	4	1,200	Poor for tinnitus and HFS; excellent for TN
18	66	F	–	16	Good	2	600	N/A
21	79	F	–	39	Excellent	2	300	N/A
22	63	F	Tinnitus	8	Excellent	1	600	Good
23	64	F	–	52	Excellent	2	300	N/A
24	59	F	Tinnitus	48	Excellent	1	300	Excellent
25	55	M	–	35	Excellent	1	300	N/A
26	67	M	Tinnitus, TN	36	Excellent	1	300	Excellent for both
27	76	M	–	34	Excellent	2	600	N/A
28	57	M	Tinnitus	42	Excellent	1	1,200	Modest
29	72	M	Tinnitus	23	Excellent	2	300	Good

HFS, hemifacial spasm; TN, trigeminal neuralgia; VP, vestibular paroxysmia.

### Therapeutic response for concomitant typewriter tinnitus and other paroxysmal cranial nerve disorders

Of the patients with concomitant typewriter tinnitus, 12 patients were treated with oxcarbazepine (Table 3) and one was treated with gabapentin. Therapeutic response for tinnitus at the stable dosages for dizziness were excellent in three patients (23.1%), good in 5 (38.5%), modest in 4 (30.8%) and poor in 1 (7.7%). The therapeutic responses for TN (*n* = 2) were both “excellent” while the responses for HFS (*n* = 2) were both “poor.” An optimal therapeutic dose range for these poorly-responsive tinnitus and HFS symptoms was not clear because clinicians targeted at the most bothersome vestibular symptoms of these patients and did not necessarily up-titrate the dosages to treat other concomitant paroxysmal cranial nerve disorders.

### Remission

**Figure 2:**
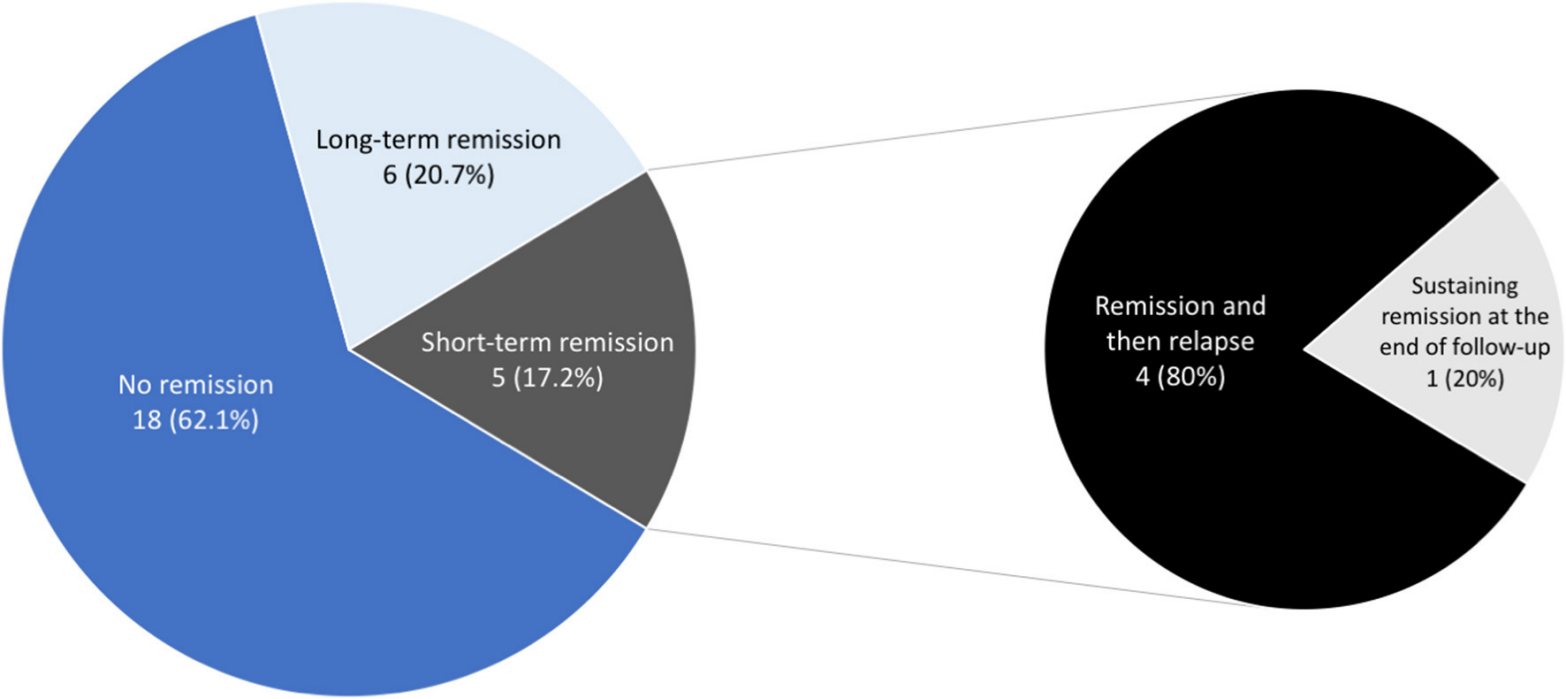
Short-term and long-term remission off medication.

**Figure 3:**
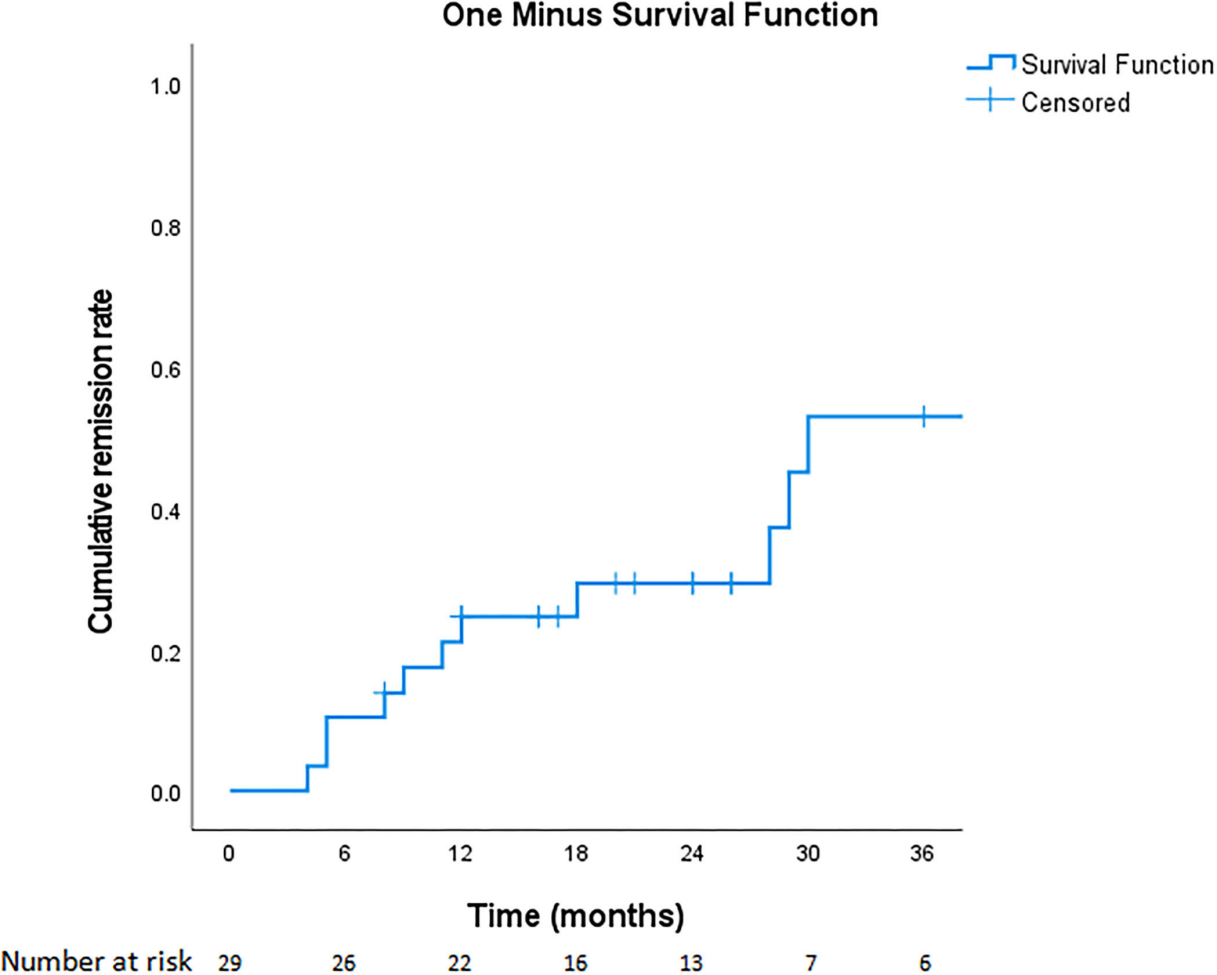
Cumulative remission rate since medication treatment for VP.

Overall, 11 of 29 patients (37.9%) experienced various periods of complete remission without medication, including 6 (20.7%) with long-term remission and 4 (13.8%) who experienced short-term remission but subsequent relapse (Figure 2). Another patient (3.4%) had been in remission off medication at 6 months (which coincided with the end of the study, so we cannot for sure say the remission was “long term”). Regarding the four who achieved short-term remission, these patients essentially relapsed within 4–8 months and had to restart medical treatment. The six patients with long-term remission had been symptom-free off medication for at least 20–43 months, as confirmed by follow-up telephone interviews. The duration of VP, the age at diagnosis, the presence of NVC on MRI, the presence of concomitant paroxysmal cranial nerve disorders, and the duration of treatment did not predict short-term or long-term remission (Table 4). Since the duration of follow-up varies in each patient, we demonstrate the cumulative remission rate considering censored data in Figure 3.

**Table 4:** Univariate logistic regression analysis for different variables for predicting remission.

**Variables**	**OR for remission**	**95% CI**
Duration of VP	0.98	0.95–1.02
Age at diagnosis	0.95	0.87–1.05
NVC on MRI	3.94	0.63–24.73
Concomitant paroxysmal cranial nerve disorders	2.63	0.53–13.07
Duration of treatment	0.96	0.91–1.01

NVC, neurovascular compression; VP, vestibular paroxysmia.

## Discussion

There are two important findings of this study in long-term outcome (>6 months) after medical treatment for VP. First, we found that complete remission off anticonvulsants occurs in about 40% of VP patients, and about 20% of these patients remain symptom-free for more than 1 year after discontinuing their medication. To our knowledge, this phenomenon has not been well-documented in the literature. Second, most patients (93.1%) report good-to-excellent therapeutic effect within 2 weeks of oxcarbazepine treatment, and most (84.6%) remain well-controlled at a low dosage of oxcarbazepine (i.e., 300–600 mg/day) in long-term follow-up.

The underlying pathophysiology of VP is assumed to be microvascular compression of the eighth cranial nerve. We were somewhat surprised to see such a high rate of remission (37.9%) because one might not expect medical treatment (sodium channel blockade) to physically relieve NVC or restore myelination. However, a similar finding has been shown in another study with 73 definite VP patients, of which 56% became attack-free after discontinuing carbamazepine or oxcarbazepine (10). In clinical practice the possibility of remission in some TN patients has been suspected for years (4). A large prospective study of TN showed that 63% of cases experienced remission for at least 1 month. Pain-free periods of several years were not uncommon (11). Over time, partial remyelination, or normalization of membrane-channel activation may reduce nerve excitability, bring the triggering level below threshold, and result in remission of TN (5). The same mechanism may, at least partially, explain our finding in the present study on VP. Moreover, some of our patients did not demonstrate evident NVC on MRI, which is similar to the condition in idiopathic TN. Positive therapeutic response of anticonvulsants in VP supports the pathomechanism of ectopic/ephaptic discharges, whether NVC is present or not. The role of NVC in VP warrants further clarification.

In our study, response to VP-specific medication was almost stable and long-lasting. This suggests only very selected patients should be considered for microvascular decompression surgery. Moreover, given the number of our patients with long periods of remission of medication, this study hints at the possibility that a drug taper can be attempted after patients have achieved an initial satisfactory response. Though some patients will still require continuation at the lowest effective dose, a large enough proportion of patients achieved remission in our study that tapering should be considered. More work is needed to confirm these observational findings.

Although the ICVD criteria include a positive response of sodium channel blockers to support the diagnosis of definite VP, studies about the long-term efficacy of oxcarbazepine are still limited (12, 13). Hufner et al. first demonstrated the effectiveness of oxcarbazepine in five cases of VP in 2008 (12). A retrospective analysis of 196 cases of VP in 2016 only looked at short-term (12 week) treatment effect of adjuvant betahistine (14). A randomized, cross-over trial showed a significant benefit of oxcarbazepine over placebo, but the high dropout rate in the trial resulted in only using data of 18 patients for analysis (15). More recently, there were two studies investigating the treatment outcome of VP. One study included 63 patients with carbamazepine but only 10 patients with oxcarbazepine. Most of them had good treatment responses and outcomes (10). The other study concluded an unfavorable outcome, but there were only 12 patients treated with carbamazepine and none with oxcarbazepine (16). When compared with these previous studies, our study is the largest case series of long-term oxcarbazepine monotherapy for VP in the real world.

In our study, most patients (93.1%) reported good-to-excellent improvement within the first 2 weeks of medical therapy. This finding also has diagnostic value. If a patient fails to show improvement on oxcarbazepine in 2 weeks, VP may not be the correct diagnosis. We also found that most patients (84.6%) obtained satisfactory results at a stable dosage of oxcarbazepine 300–600 mg/day while only four patients (15.4%; two with concomitant HFS) needed a dosage higher than 600 mg/day. As a result, one should expect to see significant improvement in most patients at a dosage of oxcarbazepine 600 mg/day. Patients who fail to show any improvement at this dosage should be considered to have other etiologies of dizziness. Our proposed dosing plan is listed in Supplementary Table 2.

Besides, our study illustrated the treatment results of anticonvulsants on concomitant typewriter tinnitus. In a literature review of combined VP and typewriter tinnitus, only 5 of 21 (23.8%) cases were able to achieve complete remission of tinnitus by drug therapy alone (17). In our cohort, only 8 of the 13 (61.5%) patients reported good or excellent responses in tinnitus, despite all of them having good-to-excellent responses in terms of dizziness symptoms. Both studies suggested that the control of auditory symptoms was suboptimal at stable dosages required for control of vestibular symptoms. We speculate that the dosage required to relieve auditory symptoms is higher than that required to relieve vestibular symptoms. The differential drug response between these two symptoms may result from the difference in nerve sizes and nuclei reorganization (18). Accordingly, our findings suggest a higher dosage of oxcarbazepine or an add-on anticonvulsant may be necessary to fully relieve the tinnitus.

Interestingly, our study revealed that only 65.5% of VP patients had the evidence of NVC in MRI, which was lower than the previous reports (12, 13, 19, 20). To our knowledge, NVC being the cause of VP is still debated given that up to 25% of healthy people have NVC (21). In our study, the patients with NVC did not show different therapeutic response or remission rate when compared to those without NVC. More importantly, in the 13 patients with combined VP and typewriter tinnitus, only seven had the NVC which were ipsilateral to the side of tinnitus. Therefore, our study findings do not fully support the theory of NVC. In our opinion, when the clinical presentations are compatible with VP, the patients should be treated with VP medication first even though their MRI findings are negative.

Some limitations of this study should be addressed here. Firstly, this was a retrospective study. Two factors may have unduly influenced our remission rate: (i) duration of follow-up differed among patients, and (ii) and some patients were more hesitant than others to discontinue medications for fear of relapse—even though they remained symptom-free on medication. In addition, to meet diagnostic criteria for VP, all patients in this study had to already show positive therapeutic response to carbamazepine or oxcarbazepine (i.e., positive drug response is part of the diagnostic criteria for VP). Therefore, we did not include the patients who potentially had NVC-induced vertigo but were resistant to these two medications. Additionally, metrics on dizziness frequency were self-reported and thus subject to recall bias. We also did not use formal scales for assessing the severity of dizziness, such as the Dizziness Handicap Inventory. Lastly, none of our patients received carbamazepine so we did not compare the efficacy of carbamazepine and oxcarbazepine.

## Conclusion

Vestibular paroxysmia is a debilitating but treatable condition. In this study, medical treatment for VP remains remarkably effective even when patients are followed longitudinally. Moreover, a significant number of patients see complete remission off medication, supporting the notion that medication taper can be considered in select cases. Given the long-term stable response to medication, surgical microvascular decompression should be reserved only for truly refractory cases. In the future, a randomized controlled trial is needed for further confirming the long-term efficacy and outcome of drug treatment for VP.

## Data availability statement

The original contributions presented in the study are included in the article/Supplementary material, further inquiries can be directed to the corresponding author/s.

## Ethics statement

The studies involving human participants were reviewed and approved by Taipei Medical University Joint Institutional Review Board (N202108060). Written informed consent for participation was not required for this study in accordance with the national legislation and the institutional requirements.

## Author contributions

C-CC designed the study, collected, analyzed, and interpreted the data, drafted the manuscript, and approved the final manuscript. T-YL and H-HL collected the data and designed the tables, and approved the final manuscript. Y-HK performed statistics and analyzed the results. AB revised the manuscript and approved the final manuscript. T-PC designed the study, interpreted the data, revised the manuscript, and approved the final manuscript. All authors contributed to the article and approved the submitted version.

## Funding

This research was supported by Taipei Medical University (TMU106-AE1-B07).

## Conflict of interest

The authors declare that the research was conducted in the absence of any commercial or financial relationships that could be construed as a potential conflict of interest.

## Publisher's note

All claims expressed in this article are solely those of the authors and do not necessarily represent those of their affiliated organizations, or those of the publisher, the editors and the reviewers. Any product that may be evaluated in this article, or claim that may be made by its manufacturer, is not guaranteed or endorsed by the publisher.
