# Supplementary material for: Vestibular paroxysmia: Long-term clinical outcome after treatment
Source: Front Neurol. 2022 Oct 14;13:1036214. doi: 10.3389/fneur.2022.1036214 (PMC9614226; doi:10.3389/fneur.2022.1036214)
Supplement: Supplementary file 1 [file Table_1.pdf]

eTable 1: Detailed data of VP cohort

No.	Age	Sex	Duration of symptoms	Concomitant NVC disorders	Medication	Stable drug dosage (mg/d)	Duration of drug treatment (months)	Duration of follow-up (months)	Active drug treatment at follow-up	Remission
1	54	M	1 year	-	OXC	300	10	14	Yes	4 months (with relapse)
2	65	M	2 months	Tinnitus	OXC	300	34	42	Yes	8 months (with relapse)
3	71	F	1 year	Tinnitus	OXC	600	36	44	Yes	8 months (with relapse)
4	72	M	4 months	Tinnitus	OXC	300	44	50	Yes	6 months (with relapse)
5	76	M	1 month	-	OXC	300	15	15	Yes	-
6	73	M	5 years	-	OXC	300	10	10	Yes	-
7	57	M	2 years	Tinnitus	OXC	900	25	25	Yes	-
8	69	M	Few years	Tinnitus	OXC	300	22	22	Yes	-
9	48	M	Few years	-	OXC	300	14	14	Yes	-
10	57	F	4 years	Tinnitus	OXC	450	56	56	Yes	-
11	66	M	1 month	-	OXC	600	44	44	Yes	-
12	73	M	9 years	-	PGB	150	38	38	Yes	-
13	51	M	2 months	HFS	OXC	900	42	42	Yes	-
14	69	M	Few months	-	OXC	300	33	33	Yes	-
15	74	M	5 years	-	OXC	300	14	14	Yes	-
16	73	F	5 months	Tinnitus	OXC	600	46	46	Yes	-
17	75	M	1 year	-	OXC	300	8	8	Yes	-

No.	Age	Sex	Duration of symptoms	Concomitant NVC disorders	Medication	Stable drug dosage (mg/d)	Duration of drug treatment (months)	Duration of follow-up (months)	Active drug treatment at follow-up	Remission
18	79	F	2 years	Tinnitus, HFS, TN	OXC	1200	16	16	Yes	-
19	66	F	3 months	-	OXC	600	28	28	Yes	-
20	45	F	3 years	-	PGB	37.5	20	20	Yes	-
21	44	M	3 years	Tinnitus	GBP	600	39	39	Yes	-
22	79	F	Few years	-	OXC	300	8	8	Yes	-
23	63	F	1 month	Tinnitus	OXC	600	32	52	No	20 months
24	64	F	1 year	-	OXC	300	4.5	48	No	43 months
25	59	F	1 month	Tinnitus	OXC	300	7	35	No	28 months
26	55	M	5 year	-	OXC	300	4	36	No	32 months
27	67	M	1 month	Tinnitus, TN	OXC	300	7	34	No	27 months
28	76	M	1 month	-	OXC	600	4	42	No	38 months
29	57	M	5 years	Tinnitus	OXC	1200	17	23	No	6 months

GBP gabapentin; HFS hemifacial spasm; NVC: neurovascular conflict; OXC oxcarbazepine; PGB pregabalin; TN trigeminal neuralgia; VP vestibular paroxysmia
