# Supplementary material for: Vestibular paroxysmia: Long-term clinical outcome after treatment
Source: Front Neurol. 2022 Oct 14;13:1036214. doi: 10.3389/fneur.2022.1036214 (PMC9614226; doi:10.3389/fneur.2022.1036214)
Supplement: Supplementary file 2 [file Table_2.pdf]

eTable 2: Proposed oxcarbazepine dosing plan for vestibular paroxysmia

- Starting treatment: 300-600 mg/day in a single or divided dosage
- Initial follow-up: 1 or 2 weeks later
- Titration: 150-300 mg/day increments every 1-2 weeks if response is inadequate
- Goal: more than 50% improvement (most patients can expect the effect by week 4)
- Maintenance treatment: usually 300-1200 mg/day
- Monitoring possible side effects, particularly skin rash, hyponatremia, leukopenia and elevated hepatic enzymes
- Considering other vestibular diagnoses if drug effect remains marginal by week 4
